# Ionomycin Treatment Renders NK Cells Hyporesponsive

**DOI:** 10.1371/journal.pone.0150998

**Published:** 2016-03-23

**Authors:** Gema Romera-Cárdenas, L. Michael Thomas, Sheila Lopez-Cobo, Eva M. García-Cuesta, Eric O. Long, Hugh T. Reyburn

**Affiliations:** 1 Centro Nacional de Biotecnología, Consejo Superior de Investigaciones Científicas, CNB-CSIC, Madrid, Spain; 2 Laboratory of Immunogenetics, National Institute of Allergy and Infectious Diseases, National Institutes of Health, Rockville, United States of America; The Ohio State University, UNITED STATES

## Abstract

Natural killer cells are cytotoxic lymphocytes important in immune responses to cancer and multiple pathogens. However, chronic activation of NK cells can induce a hyporesponsive state. The molecular basis of the mechanisms underlying the generation and maintenance of this hyporesponsive condition are unknown, thus an easy and reproducible mechanism able to induce hyporesponsiveness on human NK cells would be very useful to gain understanding of this process. Human NK cells treated with ionomycin lose their ability to degranulate and secrete IFN-γ in response to a variety of stimuli, but IL-2 stimulation can compensate these defects. Apart from reductions in the expression of CD11a/CD18, no great changes were observed in the activating and inhibitory receptors expressed by these NK cells, however their transcriptional signature is different to that described for other hyporesponsive lymphocytes.

## Introduction

Natural Killer cells are an important component of cancer and virus immunosurveillance. However, NK cells isolated from human patients with different types of cancer or infections, often present a reduced ability to kill or secrete cytokines in response to target cells. These observations, first made in 1976 [1], have been confirmed in multiple later studies. The recognition of tumours or infected cells by NK cells depends on a balance of signals originated from the binding of sets of activating and inhibitory receptors to their ligands expressed on target cells, so that when there is an excess of activating signals over inhibitory signaling, that exceeds a threshold for NK cell responsiveness, the NK cell responds to the target by mediating cytotoxicity and cytokine secretion [2]. This threshold for NK responsiveness acts as a checkpoint that allows NK cells to adapt their effector capacity to their host [3] and is established during development, while NK cells acquire receptor expression and become self-tolerant.

Different, but not mutually exclusive, theories have been proposed to explain NK cell education. The arming model proposes that inactive NK cells become licensed or "armed" upon interaction with inhibitory ligands. Alternatively, the disarming model postulates that functional NK cells are rendered less functional if the activating signal is not countered by inhibitory signals, while the Rheostat theory proposes that the responsiveness of the NK cell population is variegated and depends on the receptors expressed by each cell [4]. Interactions between MHC class I molecules and inhibitory receptors are known to be critical for NK cell licensing [5]. However, the threshold for responsiveness is not fixed during development, and mature NK cells can also ‘tune’ their capability to react, as demonstrated in various NK cell transfer experiments in mice [6–9]; and in humans, as observed in mismatched KIR hematopoietic stem transplantation (HSCT) for leukemia treatment [10–12], where the reactivity of NK cells can be “tuned” up or down. Moreover, NK cell responsiveness can also be downmodulated by chronic exposure to activating ligands, as has been described in multiple models, both *in vitro*, after a prolonged incubation of NK cells with target cells (*e*.*g*. [13]), and *in vivo* in mouse models where there is continuous transgenic expression of ligands for activating receptors or a chronic tumour burden (*e*,*g*. [14]). NK cells isolated from human patients with different types of cancer or infections, often also display a reduced functionality (*e*.*g*. [15–17]). It is tempting to speculate that these alterations in NK cell responsiveness after chronic exposure to activating ligands could be related to the modulation of responsiveness during NK cell licensing/education [18], and NK cell induced hyporesponsiveness could be understood as a disarming or re-education process similar to the disarming of NK cells during licensing. Little is known about the molecular bases underlying any of these processes in NK cells. Thus, a better understanding of the mechanisms regulating the threshold of NK responsiveness in disease and NK cell education is required.

For CD4^+^ T cells, the induction of unresponsiveness (anergy) has been related to alterations in cellular calcium levels and treatment with calcium ionophores such as ionomycin has proven to be a useful source of insights into the mechanisms underlying CD4^+^ T cell anergy [19]. We now report that overnight stimulation of primary human NK cells with ionomycin induces a hyporesponsive state in these NK cells that can be compensated by treatment with interleukin 2. This loss of NK cell responsiveness was not accompanied by major changes in the expression of most NK cell receptors, with the exception of CD11a/CD18, but the transcriptional signature of these ionomycin-treated hyporesponsive NK cells was distinct from those described for other hyporesponsive lymphocytes. Further characterization of this model system might provide new clues to the molecular basis of induced NK cell hyporesponsiveness. Stimulation through synergizing combinations of NK cell receptors induce fluxes of free Ca^2+^ in NK cells[20], thus ionomycin triggered calcium mobilisation could mimic an imbalanced or excessive stimulation. Moreover, recently it has been proposed that defects in the MAPK/ERK pathway are involved in NK cell desensitization [21, 22] further supporting the use of ionomycin treatment as a strategy to induce NK cell hyporesponsiveness.

## Material and Methods

### NK cell isolation

NK cells were isolated from buffy coats, donated by healthy volunteers (Regional Blood Transfusion Centre, Madrid). These experiments were approved by local ethical committees and the experiments were conducted according to the principles expressed in the Declaration of Helsinki. Peripheral blood mononuclear cells (PBMC) were isolated using Ficoll-Paque (GE Healthcare) gradient centrifugation. NK cells were then isolated and purified from PBMCs by negative selection using a magnetic bead isolation kit (Human NK isolation kit, Miltenyi Biotech).

After isolation, NK cells were expanded by culture in the presence of irradiated Daudi, RPMI-8866 and autologous PBMCs as feeder cells [23] in RPMI 1640 medium (Lonza) supplemented with 10% FBS, 10% Human Serum (Sigma Aldrich), 4 mM L-glutamine, 0.1 mM non essential amino-acids, 10 mM HEPES, 1 mM sodium pyruvate, 100 U/mL penicillin, 100 U/mL streptomycin, 50 μM ß-mercaptoethanol and 50 U/mL rhIL-2 (Peprotech).

### Cell lines

The cell lines RPMI 8866 [24], Daudi [25], K562 [26], P815 [27] and Jurkat [28] (all gifts of Prof. J.L. Strominger, Harvard University) were maintained in RPMI medium and used as target cells for human NK effectors as has been previously described [29]. The 293T cells (purchased from ECACC, Cat. No 12022001) were cultivated in Dulbecco's Modified Eagle medium (DMEM). All media were supplemented with 10% heat-inactivated FBS, 2 mM L-Glutamine, 0.1 mM non-essential amino-acids, 10 mM HEPES, 1 mM sodium pyruvate, 100 U/mL penicillin, 100 U/mL streptomycin and 50 μM β-mercaptoethanol. All cell lines were maintained at 37°C and 5% CO_2_ in a humidified incubator and split as necessary.

### Ionomycin treatment of NK cells

NK cells were washed and resuspended at 1x10^6^ cells/mL in RPMI 10% FBS (without human serum or IL-2). Cells were then treated with either 1 μM Ionomycin (Sigma) or DMSO (Sigma), as vehicle control, and cultured for 16 hours. Control and ionomycin-treated cells were then washed and allowed to rest for 24 hours at 2x10^6^ cells/mL in RPMI 10% FBS. Where indicated, during this rest day, cell cultures were supplemented with 50 U/mL IL-2 (Peprotech), 10 ng/mL IL-12 (Peprotech), 10 ng/mL IL-18 (MBL),100/1000 U/mL IFN-α (Peprotech), 1000 U/mL IL-4 (Peprotech) or 10 ng/mL IL-15 (Peprotech) (S1 Fig).

### Flow cytometry analysis

Live NK cells were stained, on ice, with fluorescently labeled or unlabeled antibodies followed by PE or FITC labeled F(ab’)_2_ fragments of goat anti-mouse immunoglobulin (Dako). For staining, cells were washed and incubated in a PBS/0.5% (w/v) Bovine serum albumin / 1% (v/v) Fetal bovine serum / 0.1% Sodium azide buffer (PBA) with the antibodies specified in each particular experiment using the amounts recommended by the manufacturer. After staining, cells were washed twice with PBA buffer and maintained on ice until analysis.

For intracellular staining, cells were first stained with antibodies on ice, washed with PBA and then fixed using 2% para-formaldehyde (PFA) fixation buffer for 10 minutes at room temperature in the dark. After fixation, cells were washed twice with PBA, and resuspended in PBA containing 0.5% Saponin (permeabilization buffer) and incubated with the desired antibodies at room temperature and in the dark. When the staining was finished, cells were washed twice with permeabilization buffer and once with PBA.

The various antibodies used are described in S1 Materials and Methods.

### Degranulation assay

NK cell degranulation was analyzed following the 2 hour protocol described in [30]. NK cells were co-incubated with K562 target cells (or other target cell lines when indicated) for 2 hours in round bottom 96-well plates, in an effector to target cell (E:T) ratio of 1:2 (generally 100,000 NK cells and 200,000 targets were used). The co-culture was harvested and stained with anti-Lamp1*APC and anti-CD56*PE antibodies for 30 minutes and the percentage of CD56^+^—Lamp1^+^ cells quantified by flow cytometry. Where indicated the cells were stimulated with PMA 100 ng/ml (Calbiochem) / ionomycin 0.5 μM (Sigma) as a positive control.

### Cytotoxicity assay

Cytotoxicity was assessed by the analysis of Propidium Iodide (PI) (Beckman) positive K562 or Jurkat cells, after co-culture with NK cells following the protocol described in [31]. Briefly, target K562 or Jurkat cells were stained with CFSE (Invitrogen) or PKH2 (Sigma) green membrane dye following the recommendations of the manufacturer. Target cells were then co-cultured with NK cells at a 3:1 E:T ratio for 1 h, 3 h or 5 hours in 96-well round bottom plates (generally 250,000 NK: 75,000 Targets were used). Target cells and effector cells cultured alone were used to measure spontaneous cell death. The co-cultures were collected and stained with 100 μg/mL PI during 10 minutes. Samples were analyzed by flow cytometry. Green only positive cells represent live target cells, while Green/PI double positive cells were scored as dead target cells.

For quantifying target cell death the following formula was used: % target cell death = 100 x (% Green^+^ & PI^+^ cells) / (% Green^+^)

### Redirected antibody dependent cytotoxicity

P815 cells were co-cultured with NK cells in the presence of different combinations of antibodies at 5 μg/mL for 2 hours, following the “Degranulation assay” protocol described previously, or 1, 3 and 5 hours, following the “Cytotoxicity assay” protocol. P815 cells loaded with isotype control antibodies and P815 cells that had not been incubated with antibody were used as controls. Before staining with anti-CD56*PE and anti-Lamp1*APC antibodies, cells were blocked with 10% mouse serum. Antibody clones used for redirected lysis assays with P815 cells are described in S1 Materials and Methods.

### Assay of IFN-γ production

IFN-γ production was analyzed by flow cytometry following the 6 hour protocol described in [30]. NK cells were co-incubated with target cells for 6 hours in a 96 well round bottom plate, in a E:T ratio of 1:2 (generally 150,000 NK: 300,000 Targets were used), in the presence of 2.5 μM Monensin (Sigma). The cells were then fixed with ice cold 2% para-formaldehyde (PFA), washed, permeabilized with 0.5% Saponin (permeabilization buffer) and stained with PE labeled anti-IFNγ antibodies.

### Conjugate formation assay

The formation of stable conjugates between K562 target cells and NK cells was analyzed using a previously described two color flow cytometry method [32], where K562 target cells were stained with the green fluorescent dye PKH2 (Sigma) while NK cells were labeled with the red dye PKH26 (Sigma), following the recommendations of the manufacturer. Control samples containing NK and target cells alone were also fixed. One NK-Target cell co-culture control tube was kept at 4°C during the length of the experiment and fixed when the experiment was finished. Finally, the PKH26 and PKH2 double positive events were quantified as conjugates, and the percentage of NK cells forming conjugates calculated as follows: % conjugates = 100 x (% PKH2^+^ PKH26^+^ cells) / (% PKH26^+^)

### Confocal microscopy

NK cells alone or NK cells co-cultured with CMAC-labelled K562 cells (1:1 ratio) were allowed to adhere to poly-L lysine coated coverslips for 1 hour. Cells were then fixed with 4% PFA and permeabilized with PBS containing 0.3% Triton X-100. Samples were blocked with 10% Human Serum (HS) and stained with primary antibodies diluted in 1% HS-PBS or 1% HS-PBS-0.3% Triton for 45 minutes at RT, washed and stained with secondary antibody. F-actin was visualized using Phalloidin-Alexa Fluor 488 (Invitrogen) incubated together with the secondary antibodies. The secondary antibodies used were GAM-Alexa Fluor 568, GAM-Alexa Fluor 488, GAM-Alexa Fluor 648, GAR-Alexa Fluor546 all from (Invitrogen). Coverslips were mounted using 15μL Prolong-Gold Antifade Reagent (Invitrogen) and allowed to air dry at room temperature and in the dark. Stained coverslips were sealed and stored in the dark at 4°C before being visualized using an Olympus Fluoview 1000 or a Leica TCS SP5 confocal microscope. Images were analyzed using ImageJ software and the Fiji image processing package [33]. LFA-1 polarization towards the immune synapse was measured by drawing areas with the "Fluorescence Ratio" plug-in for ImageJ [34] which allows calculation of the fluorescence ratio of the intensity of the signals in the synapse *vs*. non synapse membrane regions. To quantify F-actin polarization, the "Synapse measures" plug-in, which also takes into account the F-actin contributed by the target cell [35] was used.

### Gene expression analysis by microarray

After ionomycin treatment, total RNA from NK cells prepared from 4 different donors previously treated with either 1μM ionomycin or the vehicle control (DMSO) was isolated by TRIZOL reagent (Invitrogen) according to the manufacturer’s instructions, and then further purified using the RNeasy kit (Qiagen). A minimum of 4 μg of RNA was used for the gene expression analysis by microarrays. RNA quality was assessed using a Bioanalyzer2100. cDNA was synthesized and labeled with Cy3 or with Hyper5 Mono NHS ester (DMSO treated cells vs. ionomycin treated cells). The whole genome transcriptional profile was determined by hybridization with the two color AGILENT human gene expression 4x44K v2 microarray kit (Agilent G4845A) which analyzes the expression of 27,958 genes. Data were collected, and preprocessed for further analysis. Finally data were visualized using the FIESTAviewer v.1.0 software (http://bioinfogp.cnb.csic.es/tools/FIESTA/index.php). Differentially expressed genes were selected from the FIESTAviewer when they show a False Discovery Rate (FDR) < 0.05 by Rank Products analysis, and a Fold Change < = -1.6 (repressed genes) or > = +1.6 (induced genes). Microarray data were deposited in the GEO database (GSE79118) and followed MIAME requirements.

A selection of genes were analyzed by qRT-PCR to validate the microarray data.1μg of total NK cell RNA was isolated using TRizol (Invitrogen), was reverse-transcribed using random hexamers (Roche) and 100 U Superscript II RTase (Invitrogen), according to the manufacturer’s instructions. qPCR reactions were carried using Fast Plus EvaGreen^®^ qPCR Master Mix (Biotium) in a final volume of 8 μL. Each gene was analyzed in triplicate and purified water was included as a non-template control. The primers used are detailed in S1 Materials and Methods. PCR conditions were as follows: 50°C for 10’, 95°C for 2’, 40 cycles at 95°C15” and 60°C 1’. These reactions produced single PCR amplicons of the expected length and melting temperature, as assessed by dsDNA melting curve analysis. Reactions were run with the Applied Biosystems 7900HT system. The relative expression of each gene, with respect to 18S RNA, was calculated by applying the equation 2−(ΔΔCt) to obtain the relative quantification as relative quantity (RQ).

Differentially expressed genes were subjected to deeper analysis using different online tools (including Babelomics-Genecodis, Panther, g:Profiler), and compared with published data sets of differentially expressed genes under different treatments by Venn diagrams, as indicated.

### Statistical analysis

Data are represented as the mean values, and error bars show Standard errors of the mean. Statistical analysis was done with the GraphPad software (Prism). Student’s *t*-test or Mann-Whitney test was performed to compare pairs of data. Multiple data were compared with one-way analysis of variance (ANOVA) and Tukey-Karmer post-test. When two variables were analyzed, two-way ANOVA and Bonferroni post-test were used. When the p-value < 0.05, differences were considered statistically significant. p-values are indicated by asterisk as follows:* p<0.05; ** p<0.01; *** p<0.001. Non significant differences are noted as n.s.

## Results

### Ionomycin treatment renders NK cells hyporesponsive for cytotoxicity

**Fig 1:**
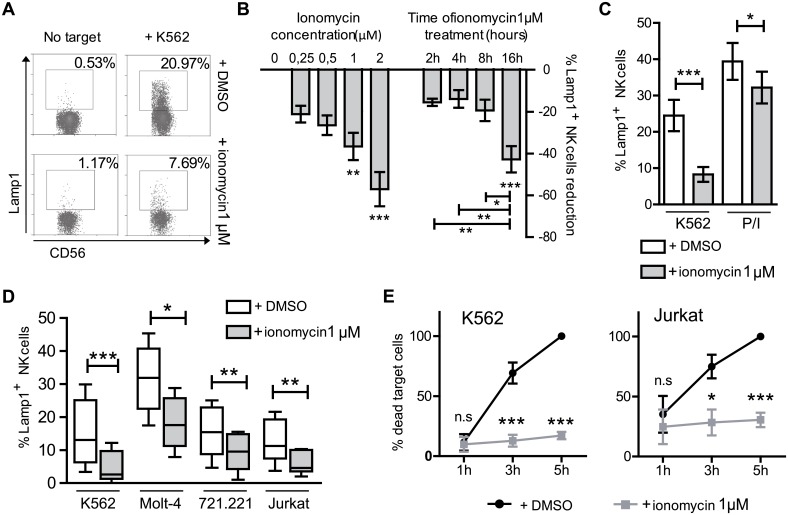
Ionomycin treatment reduces the degranulation and killing ability of NK cells. (**A**) Primary NK cells were treated with 1 μM ionomycin, or DMSO as control, during 16 hours, and after resting for 24 h, their ability to degranulate in response to K562 cells (Lamp1^+^ NK cells) was analyzed. (**B**) The induction of NK cell unresponsiveness depends on the dose of ionomycin used (0.25 μM to 2 μM) in a 16 hours treatment, and the duration of the treatment (2 to 16 hours). Data represent the percentage reduction of Lamp1^+^ NK cells in ionomycin treated cells compared to control, DMSO exposed, cells. (*n = 5*). One way ANOVA and Bonferroni post-test analysis were used to calculate statistical significance, using the logarithms of crude data. (**C**) Flow cytometry analysis shows that ionomycin-treated NK cells degranulate during 2 hours much more efficently in response to PMA/ionomycin rather than K562 cells (*n = 10*). Two tailed paired Student's T test analysis. (**D**) Ionomycin-treated NK cells respond poorly to a range of different target cells during 2 hours (*n = 6*). Two tailed paired Student's T test analysis. Whiskers show minimum to maximum data. (**E**) Reduced NK cytotoxicity measured by flow cytometry of ionomycin-treated NK cells (*n = 4*). Two way paired ANOVA analysis and Bonferroni post-test. Data show mean ± SEM (with the exception of D), *p < 0.05, **p < 0.01, ***p < 0.001.

To study the effect of ionomycin on NK cell function, human NK cells, maintained *in vitro* by weekly stimulation with feeder cells and IL-2, were washed three days post-stimulation, and exposed to 1 μM ionomycin (or DMSO, vehicle control) during 16 hours in the absence of IL-2 and human serum. Generally, some 20–30% of NK cells died during this treatment, therefore cells were washed and rested for a further 24 hours to recover before carrying out any functional assays. Initial experiments showed that ionomycin treatment rendered activated NK cells hyporesponsive to stimulation with target cells (Fig 1A). Treatment with increasing amounts of ionomycin led a gradually increasing proportion of NK cells to not degranulate in response to exposure to the target cell K562 (Fig 1B). The maximum number of cells that failed to respond was observed after 2 μM treatment, but this was accompanied by a decrease in NK cell viability (not shown), thus, further experiments were carried out using a concentration of 1 μM. Time-course experiments showed that a 16 hours treatment was needed to induce the greatest reduction in the fraction of NK cells that degranulated (Fig 1B). The induction of NK cell hyporesponsiveness after ionomycin treatment was therefore dose and time-dependent, and the need for a prolonged treatment suggests that novel protein synthesis processes are involved in the ionomycin induced NK cells loss of response. The protocol used for further experiments was as detailed in S1 Fig. Ionomycin treated cells stimulated with PMA and ionomycin for 2 hours in the absence of target cells, were still able to degranulate suggesting that the ionomycin-induced defect occurred in either, or both, target cell recognition and proximal receptor signaling. The possibility of some defect downstream of PKC and IP3 could not be completely discarded from these data since, although the difference is not statistically significant, ionomycin treated cells normally did not quite reach the level of degranulation observed for control cells after stimulation with PMA/ionomycin (Fig 1C).

Degranulation experiments after ionomycin treatment were also done using a panel of different target cells (Jurkat, Molt4 and 721.221) and similar reductions in the response of treated NK cells were observed for all of them, demonstrating that the ionomycin induced hyporesponsiveness of NK cells is target cell independent (Fig 1D). Ionomycin treatment not only reduced the ability of NK cells to degranulate, but also led to a marked reduction in NK cell cytotoxicity of two different target cell lines: K562 (where lysis is mainly dependent on lytic granules) and Jurkat cells (which express receptors for TRAIL and Fas-Ligand and thus lysis depends on also on death receptors) [36] (Fig 1E).

### Ionomycin-induced hyporesponsiveness is bypassed by IL-2 treatment

**Fig 2:**
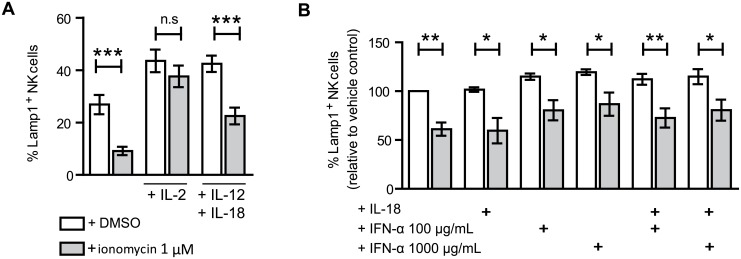
Culture in IL-2, but not IL-12 + IL18, IL-18 or IFN-α, led ionomycin treated NK cells to recover normal levels of degranulation. (**A**) NK cells were washed after ionomycin treatment, and left unstimulated, stimulated with 50 U/mL IL-2, or 10 ng/mL IL-12 plus 10 ng/mL IL-18 during the resting day (*n = 12*) Two tailed paired Student's T test analysis (**A**) or (**B**)10 ng/mL IL-18 plus 100μg/mL of 1000 μg/mL IFN-α (*n = 3*) Two way paired ANOVA analysis and Bonferroni post-test. The data show mean ± SEM. *p < 0.05, **p < 0.01, ***p < 0.001.

IL-2 treatment enhances the functionality of ionomycin induced anergic CD4^+^ T cells [19, 37]; however, previous reports were contradictory as to whether reduced NK cell responsiveness could be compensated by IL-2 stimulation [7, 13, 22, 38–40]. Culture of ionomycin treated NK cells with 50U/mL of IL-2 during the rest day re-established normal levels of degranulation on exposure to target cells implying that the effect of ionomycin imposed defect can be mitigated or bypassed by IL-2 treatment (Fig 2A).

Type 1 interferons, IL-12 and IL-18 cytokines are known to directly stimulate NK cell activity and they have been reported to also be able to rescue hypofunctional NK cells [7, 22, 38, 41, 42]. To study the influence of these cytokines in the ionomycin model, treated NK cells, were stimulated with IL-12 + IL-18, or with IFN-α, either alone, or in combination with IL-18. Although all these stimuli were able to increase NK cell degranulation in response to target cells, the difference between control and ionomycin treated cells was maintained (Fig 2B).

As the IL-2 receptor shares the common gamma chain (γc or CD132) with the receptor complexes for other cytokines including IL-15 or IL-4, it was of interest to study the effects of treatment with these cytokines on the ability of ionomycin-treated NK cells to degranulate. Stimulation with IL-15 was comparable with IL-2 in recovering NK cell functionality, in contrast to IL-4 (S2 Fig). These observations are consistent with prior data since exposure to IL-15 has long been known to enhance NK cell activity {Gamero, 1995 #559}, whereas IL-4 stimulation is known not to potentiate the cytolytic ability of human NK cells {Marcenaro, 2005 #558}.

### Ionomycin treatment affects cytokine production by NK cells, but these NK cells do not modulate the responsiveness of untreated NK cells

**Fig 3:**
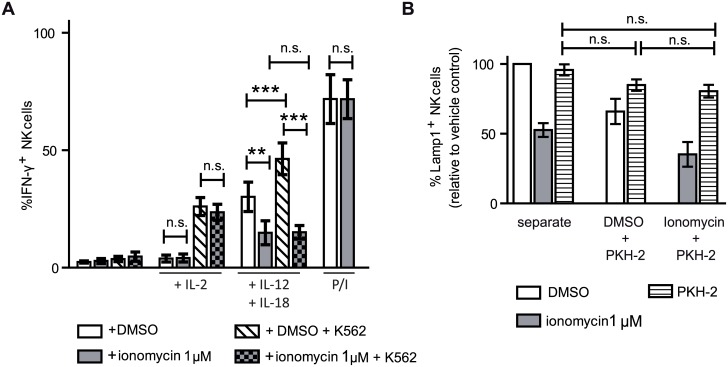
Ionomycin treated NK cells were unable to secrete IFN-γ as efficiently as control cells, and they do not secrete immunosuppressive cytokines. (**A**) IFN-**γ** production by NK cells was analyzed by flow cytometry after a 6 hours accumulation in the presence of 2.5 μM monensin. Cells were unstimulated, stimulated with 50 U/mL IL-2. or 10ng/mL IL-12 plus 10 ng/mL IL-18 during the resting day, and left alone or cocultured with K562 target cells during 6 hours. PMA/ionomycin stimulation during 4 hours of NK cells was also used to measure their potential ability to produce IFN-**γ** (*n = 6*). Two tailed paired Student's T test analysis. (**B**) Culture of autologous NK cells alone (separate) or in co-culture with DMSO control or ionomycin treated NK cells had no effect on the ability of these NK cells to respond to targets (*n = 4*). Paired one way ANOVA analysis and Tukey's multiple comparison test. The data show mean ± SEM. **p < 0.01, ***p < 0.001, *n*.*s*.: non-significant.

Another important role of NK cells is the production of cytokines and chemokines that can influence the function of other immune cells. IFN-γ production by either control or ionomycin-treated NK cells was not detected by intracellular flow cytometry after co-culture with K562 targets. Therefore, to increase the production of IFN-γ, both control and ionomycin treated NK cells were stimulated with IL-12 and IL-18 during the resting day, conditions known to enhance IFN-γ production by NK cells [43]. While IL-12/IL-18 stimulated the production of significant amounts of IFN-γ by control NK cells, ionomycin-treated NK cells produced much less. Moreover, while the production of IFN-γ by control NK cells was further increased by exposure to target cells, this effect was not observed for ionomycin treated NK cells. Similar to what was seen in the experiments of degranulation, both control and ionomycin treated cells exposed to IL-2 in the rest day were able to produce IFN-γ when exposed to target cells (Fig 3A). No difference between the percentage of IFN-γ positive cells was observed between control and ionomycin treated cells stimulated with PMA and ionomycin, however, the mean fluorescence of the ionomycin treated IFN-γ positive cells was lower (69.45% +/- 26.04% of the mean fluorescence intensity seen for the control cells treated with PMA/ionomycin) (S3 Fig), indicating that each cell produced less amount of this cytokine.

To test if ionomycin-treated, hyporesponsive NK cells could modulate the response of other NK cells, or whether culture with untreated NK cells could rescue the ionomycin-induced hyporesponsiveness, control NK cell cultures were labelled with PKH-2 dye and cultured alone or co-cultured with untreated or ionomycin-treated NK cells during their post-treatment rest day. As the degranulation ability of control PKH2-labelled NK cells was not significantly affected by co-culture with ionomycin treated cells (Fig 3B), nor was the ionomycin-induced hyporesponsiveness reversed it can be concluded that the changes underlying the hyporesponsiveness of ionomycin treated NK cells are intrinsic to the treated cell.

### Ionomycin treatment is accompanied by changes in integrin expression and function

**Fig 4:**
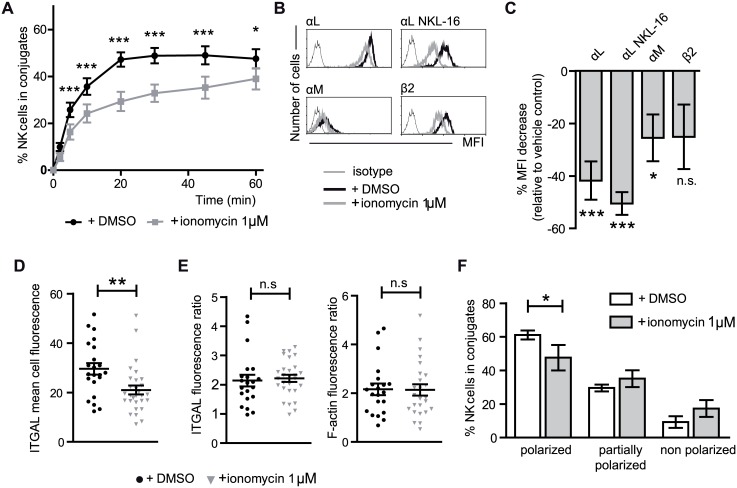
Ionomycin treated NK cells form fewer conjugates with target cells, and express lower levels of integrin molecules. However, those cells that form conjugates are able to polarize αL, lytic granules and accumulate F-actin in the immune synapse with target cells as efficiently as control cells. (**A**) NK cells and target cells were stained with different color dyes and the double positive events, viewed by flow cytometry, used to calculate the percentage of NK cells forming conjugates over time (*n = 15*). (**B**) Representative flow cytometry histograms of close and open αL, αM and β2 integrin subunits expression by flow cytometry. (**C**) Relative decrease of the MFI expression measured by flow cytometry of the different integrin molecules relative to the expression on control cells (*average values of at least 10 experiments*). Two tailed paired Student´s T test analysis of the logarithm of raw data. (**D**) Mean fluorescence intensity of LFA-1 staining in NK cells (confocal microscopy images quantitated using Image J (*n = 22* DMSO, *n = 29* ionomycin). Mann Whitney test. (**E**) Those ionomycin-treated NK cells that form conjugates with target cells are able to polarize αL and accumulate F-actin in the immune synapse. The ratios of αL and F-actin fluorescence between the synapse, and non-synapse membrane zones of control and ionomycin treated NK cells were quantified using ImageJ-FIJI (data from three independent experiments. *n = 22* DMSO, *n = 29* ionomycin). Mann Whitney test. (**F**) Lytic granules polarization on NK cells that formed conjugates by confocal microscopy was stratified in polarized, partially-polarized and non-polarized visually (data from four independent experiments. *n = 249* DMSO, *n = 97* ionomycin). Paired two way ANOVA analysis and Bonferroni posttest. The data show mean ± SEM. *p < 0.05, **p < 0.01, ***p < 0.001, *n*.*s*.: non-significant.

Ionomycin treated NK cells were ineffective mediators of both cytotoxicity and cytokine production, establishing the existence of defects in their function, but leaving open which stage(s) of the target cell recognition or effector processes were defective. After NK cell/target cell contact and recognition, the interaction between these cells is stabilized initially in a process that depends on adhesion molecules such as integrins and later involves strong binding after activation of these integrins by inside-out signaling. The ability of ionomycin treated NK cells to form stable conjugates with target cells was studied by flow cytometry (gating strategy shown in S4A Fig) and found to be less than control NK cells with maximum differences being observed between 20 and 45 minutes of co-incubation (Fig 4A) while at longer times the differences, although still significant, were less pronounced.

NK cells express different adhesion molecules, and lower expression of these molecules could explain the lower conjugate formation observed on ionomycin treated NK cells. The expression of the adhesion molecules αX, β1 and β3 integrin chains, CD44 and ICAM-1 glycoproteins, and CD58, did not differ significantly between control and ionomycin treated NK cells (data not shown). However, the expression of αL, αM and β2 integrin subunits were reduced on ionomycin treated NK cells in most of the donors analyzed (Fig 4B and 4C). When αL expression was assessed using an antibody specific for an active conformation (NKL16) and whose reactivity has been related to cluster-dependent increases in the avidity of αL integrins for their ligands [44], it was observed as decreased in all ionomycin treated cells analyzed (Fig 4C). Culture of the ionomycin treated cells in IL-2 led to a statistically significant recovery in the expression levels of the αM and αL molecules, although they didn’t quite reach the same levels of expression as control cells treated with IL-2 (considered as 100% for each molecule) (S5 Fig).

Given the contribution of activated LFA-1 (αLβ2) to granule polarization and actin polymerization [45], it was of interest to assay whether the reduced expression of LFA-1 in ionomycin-treated NK cells could also be associated with defects in immune synapse formation. Confocal microscopy confirmed that ionomycin treatment led to a lower level of expression of αL integrin (Fig 4D). However, many of those ionomycin treated NK cells that formed conjugates with K562 targets were able to polarize αL subunit, and accumulate F-actin in the immune synapse (Fig 4E) as measured by fluorescence ratio quantization, although the number of ionomycin treated NK cells that formed conjugates and polarised their lytic granules towards the synapse was significantly lower than for control cells (Fig 4F). Examples of confocal microscopy stainings are shown in S4B and S4C Fig.

These analyses of different steps in the NK cell:target cell interaction indicated that ionomycin treatment of NK cells leads to defects in the formation of conjugates with target cells and lower levels of LFA-1 expression, however the formation of the cytotoxic synapse and lytic granule polarization was reasonably conserved in those ionomycin-treated NK cells that were able to adhere tightly to target cells. Although these reductions in integrin expression could signify a reduced adhesion to target cells and so explain the lower levels of conjugate formation and cytotoxicity observed and why the degranulation machinery was activated when stimulated directly by PMA/ionomycin, but not by exposure to target cells, the levels of expression, especially those of the αL subunit, were still considerable, leaving open the possibility that ionomycin treated cells may also have other defect(s) that contribute to the observed defects in NK cell recognition. Consistent with this idea αL integrin expression was not reduced in hyporesponsive NK cells from 2/14 donors analyzed, indicating that although modulation of integrins may have an important role in NK cell hyporesponsiveness, ionomycin treated cells may also have other defect(s) which impair their function.

### Effect of ionomycin treatment on the expression and function of NK activating and inhibitory receptors

NK cell responsiveness is thought to be influenced by the repertoire and levels of receptors expressed. Given that the balance of signals coming from activating and inhibitory receptors determines whether NK cells are activated or not, lower levels of activating receptors or adhesion molecules, and/or higher levels of inhibitory receptors could explain the absence of target cell recognition and less stable conjugate formation.

**Fig 5:**
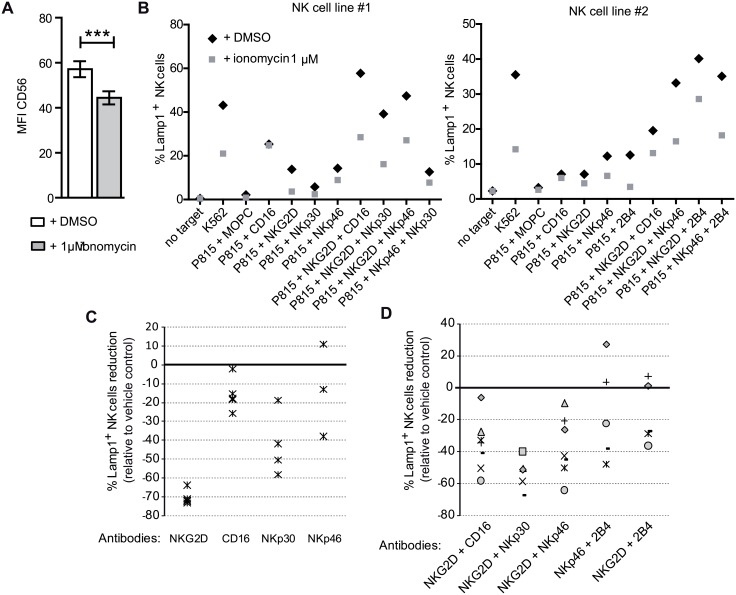
Ionomycin treatment reduces NK cell receptor function. (**A**) Ionomycin treatment reduces the expression of CD56 on NK cells. (**B**) NK cells were cocultured with P815 cells loaded with 10μg/mL of the indicated antibodies alone or in combinations. Representative examples of data from two donors. (**C**) Relative numbers of NK cells able to degranulate in response to P815 cells and the indicated antibodies (each symbol corresponds to a different experiment), or (**D**) combinations of antibodies(each symbol corresponds to a different experiment).

Flow cytometry was used to analyze the expression of NKG2D, NKp46, NKp30, CD16, CD94, 2B4, CD28, KIR2DL1/S1, KIR2DL2/DL3/S2, KIR3DL1/S1, CD94, LILRB1 (ILT2, CD85j) and CD161 (KLRB1), but no consistent difference was observed between DMSO and 1 μM ionomycin treated NK cells (data not shown). Also, no differences in expression were noted for CD28, CD45, CD53, CD59, CD40, CD69, CD86, HLA-DR, HLA-ABC, CD95 or PD1 expression (data not shown). Surprisingly, the expression of CD56 was consistently decreased (Fig 5A), however given the lack of a defined role for CD56 in NK cell biology the significance of this observation is unclear [46].

To test whether the ionomycin treatment differentially affected different NK cell populations, degranulation experiments were performed as described before, but the staining of another receptor in the analysis was incorporated into the experiment so that the Lamp1^+^ and Lamp1^−^ marker populations could be compared between control and ionomycin treated cells. Subpopulations of NK cell defined by expression of a range of molecules including (CD11b, KIR2DL1 (CD158a), KIR2DL2/DL3 (CD158b), CD16, CD57, CD8, CD69, HLA-DR and NKG2C) were analyzed. Although the number of degranulating NK cells was reduced by ionomycin treatment, the fractions of responding NK cells that expressed each marker was comparable (a representative example of these experiments is shown in S6 Fig). Thus these data argue against the hypothesis that ionomycin treatment affected a specific population of NK cells.

Although ionomycin treatment did not produce any clear differences in NK activating receptor expression it was still of interest to study the function of these molecules so redirected antibody dependent cytotoxicity experiments were performed. NK cell lines from different donors have different thresholds for activation and differential responses to different stimuli. Thus, NK cell lines from different donors differ in the cytotoxic response after activation via different receptors.

This approach allowed us to test the hypothesis that ionomycin treatment could modulate the threshold for NK cell responsiveness. When the NK cells were stimulated with single antibodies CD16 was the most potent stimulator of degranulation, and ionomycin treated cells made a response similar to that seen for control cells (Fig 5B shows experiments with two different NK cell lines). In contrast, the response of ionomycin treated NK cells to stimulation through other receptors, especially NKG2D, was much weaker than that of control cells. This experiment was repeated using NK cells from different donors and although each line showed different levels of degranulation to different receptors, the pattern of responsiveness imposed by ionomycin treatment was similar (Fig 5C). When P815 cells were loaded with combinations of antibodies specific for receptors known to synergize for NK cell activation[20], more variable results were obtained (Fig 5D each symbol corresponds to a different NK cell line), but as expected, triggered increased degranulation in both control and ionomycin treated cells compared to that observed after stimulation with a single antibody. However, the responses of the ionomycin treated cells were still lower than control cells in most of the experiments. This experiment was repeated also using multiple NK cell lines, and although each line showed different levels of degranulation, the overall pattern of a decreased responsiveness after ionomycin treatment was similar.

### Transcriptional changes in NK cells induced by ionomycin treatment

The processes of anergy [19], exhaustion [47] or tolerance [48] in T cells are reflected in changes in the expression profiles of multiple genes. As ionomycin treated NK cells exhibit functional defects, it seemed reasonable to hypothesize that these changes in function were accompanied by changes in the pattern of gene expression. Therefore cDNA microarray experiments were performed to study in detail the changes in gene expression associated with NK hyporesponsiveness. From this analysis 75 genes were found to have higher expression in ionomycin treated cells (in 4/4 donors), while 72 showed lower expression compared to control NK cells (S1 Table).

**Fig 6:**
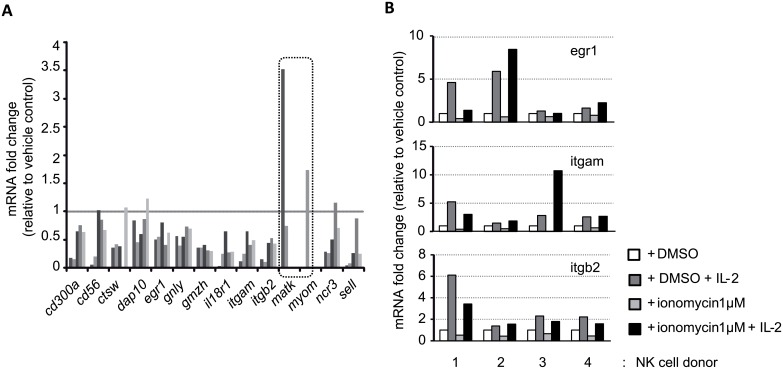
Genetic analysis of ionomycin treated NK cells. (**A**) Gene expression analysis by qPCR of 14 selected genes that appeared downmodulated in the microarray experiment comparing ionomycin treated cells to control cells. (*n =* 5). Highlighted genes represent genes that appeared downregulated in the microarray analysis, but were not confirmed by qPCR and so were not analysed further. (**B**) The effect of IL-2 on the expression, by DMSO and ionomycin treated NK cells, of the genes analyzed previously was analyzed by qPCR. IL-2 rescued the levels of expression of *egr1*, *itgam* and *itgb2* genes in all 4 samples.

To validate the microarray data the differential expression of a selection of the downmodulated genes was analyzed by qRT-PCR (*cd56*, *ctsw*, *dap10*, *egr1*, *gnly*, *gzmh*, *il18r1*, *itgam*, *itgb2*, *matk*, *myom*, *ncr3*, *sell*) using mRNA from control and ionomycin treated NK cells isolated from other 6 different donors. (Fig 6A). This qPCR analysis confirmed that most, although not all, of the genes classified as downmodulated in the microarray analysis, were downregulated in the mRNA isolated from ionomycin-treated NK cells from different donors, thus validating the data obtained from the microarray experiments. The two genes not confirmed by qPCR (*matk* and *myom2*) were not analysed further. Interestingly, although IL-2 stimulation restored *egr1*, *itgam1*, and *itgb2 mRNA* expression to levels comparable to those of control cells (Fig 6B), CD18 expression at the cell surface was still significantly less than that observed on control cells (S5 Fig).

Gene Ontology analysis suggested that several pathways or functions were enriched in the lists of genes up-regulated and downregulated after ionomycin treatment (S2 Table). The downregulated list of genes is enriched in genes related to chemotaxis, adhesion and cytotoxicity, thus it is easy to imagine that deficient expression of these genes will profoundly affect NK cell response and behavior. The upregulated list of genes is enriched in regulatory genes, phosphatases and oxidoreductases that could modulate signal transduction. The largest group of genes downmodulated by exposure to ionomycin were chemokine receptors and ligands suggesting further defects in defects in NK cell migration, tissue infiltration and activation.

To further understand the importance of the differentially expressed set of genes obtained in the ionomycin treated NK cells analysis, the gene signature obtained from these cells was compared with previously described NK cell gene expression signatures (S7 Fig). In [49] the authors compared the gene expression of human NK cells with the molecular signature of other cell types. Comparison of their gene analysis with the lists of genes altered by ionomycin, revealed that 3 ionomycin upregulated genes, appeared in their gene analysis, but 18 of the genes which define their NK cell signature were downmodulated. These data might suggest a possible NK-“dedifferentiation” process, however this suggestion is only speculation. Moreover, comparison of the ionomycin signature with those of activated CD56^dim^CD16^+^ NK *vs*. non-activated CD56^dim^CD16^+^ NK cells again identified multiple genes in common with the downregulated ionomycin signature [50].

Gene expression of ionomycin treated NK cells was also compared with the genes differentially expressed in different situations associated with hyporesponsiveness of CD4^+^ and CD8^+^ T cells (anergy, exhaustion, tolerance), however only a few genes were found with similar or inverse regulation[47, 48, 51]. Surprisingly however, no genes modulated by ionomycin in NK cells were found in common with the genes upregulated in ionomycin treated CD4^+^ T cells described by Macian *et al*. [19].

## Discussion

Although hyporesponsive NK cells have been described in patients suffering tumors or infections, little is known about the molecular changes that produce this state. In fact, many of the characteristics described for hyporesponsive NK cells in different studies are not consistent between studies, probably due to the heterogeneity of diseases, patients and models used and NK hyporesponsiveness continues to be an important topic to address. Also, the molecular mechanisms underlying NK cell responsiveness are not clearly established, and further knowledge on this subject of basic NK cell biology is of great interest.

In this work, we studied the use of ionomycin as a method to induce NK cell hyporesponsiveness. This model has the advantage of simplicity, reproducibility and as it is a non-specific stimulation, it increases the likelihood of identifying common processes underlying NK cell hyporesponsiveness independent of the patient or disease. Ionomycin treatment reduced, in a dose and time dependent manner, the ability of IL-2-cultured NK cells to degranulate in response to target cell stimulation. Treatment with ionomycin during 16 hours proved to be the optimal compromise between the reduction of function and maintenance of cell viability. Ionomycin treatment decreased the ability of NK cells to kill target cells and produce IFN-γ in response to either target cells or IL-12 and IL-18 and this cells also show a reduced ability of these ionomycin-treated cells to form conjugates with target cells. Interestingly, however, those NK cells that did adhere to target cells were able to create functional immune synapses and polarize their lytic granules. As has been described for CD4^+^T cells, culture in IL-2, led to a recovery or bypass of the activity of the NK cells rendered unresponsive by ionomycin treatment[52].

Apart from a lower expression of αL, αM and β2 integrins, no consistent differences in the expression of a wide range of NK cell surface molecules, including activating and inhibitory NK receptors were found between control and ionomycin-treated NK cells. Similarly, no differences in expression of a range of different receptors were found between the NK cell populations that did, or did not, degranulate after exposure to K562. These data argue against the idea that in ionomycin treated cells the NK cell repertoire was shaped toward the selection of NK cells with impaired cytolytic activity or that the population of cells inactivated by ionomycin treatment represents a particular subset of NK cells. Thus ionomycin could be shifting the responsiveness of the entire NK cell population.

As discussed in the introduction, a threshold for NK cell responsiveness is established during their education. This set point is not fixed, but rather is variable since mature NK cells can be re-educated during their entire lifetime. The results from the experiments on ionomycin treated NK cells suggest that ionomycin treatment shifts the NK cell activation threshold so that an increased signal intensity is necessary to mount an effective response. Assuming that the range of responsiveness of a population of NK cells follows a normal distribution this hypothesis explains why some cells maintain the ability to respond to target cell stimulation, form stable conjugates and polarize molecules to the immune synapse, despite ionomycin treatment; by contrast, another part of the population is rendered unresponsive, but able to respond when sufficiently potent stimulation is received, as with CD16 stimulation or after stimulation with cytokines such as IL-2 or IL-15. One caveat to this idea is that increasing stimulation by triggering NK cells through synergistic combinations of receptors did not overcome the effect of ionomycin treatment, however this might be explained if, apart from an altered threshold for responsiveness, ionomycin treated cells also have problems creating synergy between receptors due, for example, to changes in receptor mobility in the plasma membrane as has been reported for unlicensed NK cells [53]. Alternatively, ionomycin treatment may also affect signaling events important for synergy (eg c-Cbl signaling or SLP-76 phosphorylation [54, 55]).

This hypothesis of variation in thresholds of responsiveness for activation would also explain why although 1 μM ionomycin was sufficient to induce hyporesponsiveness in NK cell lines prepared from the majority of donors, NK lines from some donors were relatively unaffected by this dose and a larger amount of ionomycin was required to induce unresponsiveness, while others died due to the stimulation (data not shown). This model is a logical extension of the disarming and rheostat models proposed for NK cell licensing. When the balance between activating and inhibitory signals is below the threshold for response set for each NK cell, the cell would not respond. If the threshold is exceeded, the NK cell becomes activated. However, if the net balance is much higher, as could be happening in the context of chronic activating receptor stimulation, a loss of responsiveness is induced in mature NK cells [3]. NK cells would have then two thresholds, one for response, and one for inactivation due to overstimulation (S8A Fig). If the signal received exceeded the second threshold, NK cell clones would begin to become inactivated (disarming model), or the responsiveness could be readjusted downward until it is counterbalanced again, setting a higher response threshold (rheostat model). Ionomycin treatment of NK cells, can be thought of as an overstimulation signal, which instead of causing higher responses of an NK cell population, recalibrates each NK cell so that a higher activation threshold is established, so that while most cells become hyporesponsive, others would still be able to degranulate despite the threshold recalibration. Similar events might occur in a tumour or patients with chronic virus infections, where viruses or tumours activating chronically NK cells, could take advantage of this self-defensive mechanism to avoid NK cell hyperactivation, leading to an adjustment of the NK cell responsiveness and hyporesponsiveness (S8B Fig). This equilibrium could be altered by the inflammatory stimulation, and the secretion of different cytokines, chemokines or antibodies, or the interaction with other cells, could lower the NK cell threshold for response, favouring NK cell activation. This ability of NK cells to adapt to modifications in their environment is consistent with the *discontinuity* model [56]. This model proposes that immune responses are triggered when a discontinuity (an altered self) in the microenvironment is detected. Logically, however, maintenance of the activation signal would lead to a re-education towards a non-responsive state, consistent with re-education of mature NK cells after ionomycin overstimulation.

One outstanding question is the mechanism by which treatment with ionomycin alters the responsiveness of NK cells. The data to date do not provide a definitive answer to that question, however one consistent difference between NK cells treated with ionomycin or the vehicle control was a clear reduction in the levels of expression of integrins (mainly αL, αM and ß2 chain) at the cell surface of ionomycin treated NK cells (Fig 5C). The lower level of expression of integrin molecules is associated with lower levels of mRNA for the β2 integrin molecule and it seems reasonable to suggest that this might be related to the observation of a lower efficiency in the formation of conjugates between NK cells and K562 target cells (Fig 4A). Interestingly, in a murine model of β2 integrin-deficiency NK cells displayed a hyporesponsive phenotype *in vitro* [57]. Moreover, defects in conjugate formation have been described in NK cells from cancer patients [58, 59]. Integrin signaling has also been shown to have an important role in polarization of the cytotoxic granules of NK cells, and although many ionomycin treated NK cells did not adhere stably to target cells, those cells that did form conjugates were able to polarize lytic granules and LFA-1 (Fig 4E and 4F). Similar observations have been made in studies of unlicensed human NK cells that have a reduced ability to form conjugates with target cells, but normal polarization of lytic granules[60] and this phenotype has been linked with a defect in inside-out activation of LFA-1. Thus, ionomycin treatment could be targeting inside-out activation and this idea is consistent with the decreased level of reactivity with the NKL-L16 antibody observed in our experiments (Fig 4C), but ionomycin treatment could also be affecting the composition or the release of lytic granules following their polarization. Super-resolution microscopy of primary human NK cells has revealed that LFA-1 signaling is important for remodelling the meshwork of cortical actin at the synapse to favour the release of lytic granules after NK activation [61].

Analysis of the ionomycin treated NK cells transcriptome, identified several genes that had been either up- or downregulated. Comparison of these genes with transcription profiles of other types of hyporesponsive lymphocytes did not identify genes in common. However, many of the genes downmodulated after ionomycin treatment are genes whose expression is normally upregulated in the NK cell lineage, perhaps indicating a loss of the "natural killerness" of ionomycin-treated NK cells. Interestingly, the gene signature of ionomycin treated NK cells didn’t correspond strongly with the signature from other hyporesponsive cells under different circumstances, thus no potential candidate could be identified as a common gene which could regulate the responsiveness of both NK and T cells.

Ionomycin treatment has shown to be a useful tool to understand NK cell unresponsiveness, however the precise molecular basis of the modulated responsiveness remains unclear. Further studies in this model could be useful to generate hypotheses to guide future analyses of the properties of hyporesponsive NK cells.

## Supporting Information

S1 Materials and Methods List of antibodies and primers used in this study. (PDF)

S1 Fig Diagram of the protocol used to study the ionomycin induced NK cell loss of function. (PDF)

S2 Fig Culture in IL-15, but not IL-4 led ionomycin treated NK cells to recover normal levels of degranulation. During the rest period aliquots of ionomycin treated or control cells were cultured either in medium alone, or medium supplemented with the indicated recombinant cytokines: IL-2, 50U/ml; IL-4, 1000U/ml; IL-15, 10ng/ml. After this culture the NK cells were recovered, washed counted and used in degranulation assays against K562 target cells. (*n =* 3). Two way paired ANOVA analysis and Bonferroni post-test. The data show mean ± SEM. ***p < 0.001.
(PDF)

S3 Fig Ionomycin treated NK cells produce less IFN-γ per cell after PMA/ionomycin stimulation. IFN-γ production by NK cells was measured by flow cytometry after a 4 hours accumulation in the presence of 2.5 μM monensin. Data shown as mean fluorescence intensity (*n = 8*).
(PDF)

S4 Fig **(A)** Gating strategy followed to study conjugate formation between NK and K562 cells. **(B)** Representative examples of control (top) and ionomycin treated (lower) cells staining of single Alexa 648 (LFA-1) and Alexa 488 (F-actin) channel colors and their merge. **(C)** Representative example of lytic granules polarization as non-polarized, partially polarized and polarized. F-actin (Phalloidin-Alexa 488) appears in green, lytic granules in red (Perforin antibody + GAM Alexa 568I and target cells in blue (CMAC dye).
(PDF)

S5 Fig Relative decrease of the MFI of the different integrin molecules relative to the expression on control cells (White bars), Grey bars: ionomycin treated cells. Hatched grey bars: Ionomycin treated cells stimulated with IL-2 during the rest day (*n = 3–12*). The data show mean fluorescence ± SEM of those experiments where integrin expression decreased. Two tailed paired Student's T test analysis of the logarithm of raw data was used. *p < 0.05, **p < 0.01, ***p < 0.001, *n*.*s*.: non-significant.
(PDF)

S6 Fig DMSO/control or ionomycin-treated NK cells were cultured with K562 targets for 2hours and then stained for Lamp1 and either isotype-control or specific mAbs. NK cells were identified as CD56 positive cells ([B] gate), and gated as marker negative ([C] gate) and positive ([D] gate. Percentage of positive cells is shown). The degranulation of cells positive and negative for each marker analysed is also shown (percentage and geometric mean of fluorescence intensity of Lamp1 positive cells).
(PDF)

S7 Fig Analysis of genes that are either upregulated (Io-Up) or downregulated (Io-Down) after ionomycin treatment of NK cells related to other immune gene signatures. (PDF)

S8 Fig (**A**) The disarming model implies that during education there exists one threshold for inactivation, which if exceeded triggers NK cell hyporesponsiveness. When licensed, NK cells will have two thresholds, one for activation and another for inactivation after overstimulation. (**B**) Ionomycin treatment could cause a recalibration of at least the NK cell threshold for response, increasing the amount of activation signal required for an NK cell response.
(PDF)

S1 Table Up/down-modulated genes on 4 NK cells from 4 different donors treated with 1 μM ionomycin. (PDF)

S2 Table Gene ontology analysis of differentially expressed genes on ionomycin treated NK cells highlighting different pathways, cellular localizations and functions. (PDF)
