# Supplementary material for: Ionomycin Treatment Renders NK Cells Hyporesponsive
Source: PLoS One. 2016 Mar 23;11(3):e0150998. doi: 10.1371/journal.pone.0150998 (PMC4805247; doi:10.1371/journal.pone.0150998)
Supplement: S1 Materials and Methods — (PDF) [file pone.0150998.s001.pdf]

# Supporting materials and methods.

## Antibodies

Antibodies clones used for flow cytometry and, confocal microscopy are: CD2-APC (LT2, Immunotools), CD9 (MEM-192, a kind gift of Dr V.Horejsi, Institute Molecular Genetics AS AR), CD11a (HI111, BioLegend, AB_314151), CD11a open (NK1-L16, kindly provided by C.G. Figdor, Nijmegen Center for Molecular Life Sciences), CD11a-CD18 (Ab24, Abcam, ), CD11b (ICRF44, BioLegend, AB_314154), CD11b activated (CBRM1/5, Biolegend, AB_314166), CD11c-APC (BU15, Immunotools, AB_1279066), CD16 (B73.1 (a kind gift of J.L. Strominger, Harvard University), CD18 (LIA3/2 ascites, a kind gift of F. Sánchez-Madrid, H. La Princesa), CD25-FITC (HI25a, Immunotools, AB_1071798), CD27-PE (LT27, Immunotools), CD28 (CD28.2, BD Pharmingen, AB_396068), CD29-FITC (MEM-101A, Immunotools, AB_1056084), CD44-APC (MEM-85, Immunotools, AB_522565), CD45-FITC (MEM-28, Immunotools, AB_1144367), CD49d-FITC (BU49, Immunotools, AB_960490), CD53-FITC (HI29, Immunotools, AB_1083941), CD54-FITC (1H4, Immunotools, AB_1056464), CD56 unlabelled / PE (MEM-188, BioLegend, AB_314444 / AB_314447), CD58-PE (MEM-63, Immunotools, AB_300983), CD59-APC (MEM-43, Immunotools, AB_1052192), CD61-FITC (C17, Immunotools, AB_2128768), CD62L-APC (LT-TD180, Immunotools, ), CD69 (FN50, BD Pharmingen, AB_395914), CD85j (GHI/75, BD Pharmingen, AB_394020), CD94-PE (HP-3D9, BD Pharmingen, AB_396201), CD95 (IM2387, Immunotech, AB_131702), CD107a-APC (H4A3, BioLegend, AB_1279055), CD152-PE (BN13, Immunotech, AB_131390), CD158a (EB6, Immunotech, ), CD158b (GL183, Serotec, AB_2265256), CD158e1 (DX9, BD Pharmingen, AB_396258), DNAM1 (MAB666,

R&D, AB_2072624), HLA-ABC-PE (W6/32, Immunotools, AB_964521), HLA-DR-PE (LT-DR, Immunotools), IFN-γ-PE (4S.B3, BioLegend, AB_2123323), Mouse IgG1 (MOPC-21, Sigma, AB_1163685), NKG2D (MAB139, R&D, AB_2133263), NKp30 (210845, R&D, AB_2149446), NKp46 (195314, R&D, AB_2149153), Perforin (dG9, BioLegend, AB_314699), Pericentrin (4488, Abcam, AB_304461), PD1 (EH12.2H7, BioLegend, AB_940485), β-actin (AC-15, Sigma, AB_476692).

Monoclonal antibodies used to stimulate the different NK cell receptors, either individually or in combination, were 2B4 (2-69, B&D Pharmingen, AB_393899, Nakajima et al, "Activating interactions in human NK cell recognition: the role of 2B4-CD48. Eur J Immunol." 1999 29:1676-83) CD16 (B73.1, Perussia et al, "Human natural killer cells analyzed by B73.1, a monoclonal antibody blocking Fc receptor functions. II. Studies of B73.1 antibody-antigen interaction on the lymphocyte membrane." J Immunol. 1983 130:2142-8, a kind gift of J.L. Strominger, Harvard University), NKG2D (MAB139 R&D), NKp30 (210845, R&D), NKp46 (195314, R&D), and Mouse IgG1 (MOPC-21, Sigma) as a negative control.

## Primers used for qPCR

| GENE | Forward primer | Reverse primer |
|---|---|---|
| *dap10* | 5'- GCACTTCAGGCTCTTGTTC | 5'- GCCTGGCATGTTGATGTAGA |
| *itgb2* | 5'- CAAGCTGGCTGAAAACAACA | 5'- ATTGCTGCAGAAGGAGTCGT |
| *itgam* | 5'- GCCGGTGAAATATGCTGTCT | 5'- GCGGTCCCATATGACAGTCT |
| *myom2* | 5'- GAACCCCACAATTCCTCTGA | 5'- TCGATCCATCACCATCTCAA |
| *gzmh* | 5'- CAGAAGGACTGCCAGTGTGA | 5'- ACTCCTGGAGGTGTCCCTTT |
| *cd300a* | 5'- GTCCTGCAAACCTCAGCTTC | 5'- CCCACTGCAAACAGGGTAGT |
| *il18r1* | 5'- TAGTGCCTGGAGGAGCTGTT | 5'- ATTGGGGCAAGAATGTGAAG |
| *egr1* | 5'- TGACCGCAGAGTCTTTTCCT | 5'- TGGGTTGGTCATGCTCACTA |
| *ncr3* | 5'- CCTGAGATTCGTACCCTGGA | 5'- GAAACGGGAAGAAGCAAGTG |
| *ctsw* | 5'- CCACCCCAAGAAGTACCAGA | 5'- GTGGCCTTGATCACACCTTT |
| *sell* | 5'- AAACCCATGAACTGGCAAAG | 5'- CGCAGTCCTCCTTGTTCTTC |
| *matk* | 5'- TACCGCGTCAAGCACCACACCAG | 5'- CACTCTCCACTCTCTCGGTCCTCTG |
| *cd56* | 5'- TATTTGCCTATCCCAGTGCC | 5'- CATACTTCTTCACCAACTGCTC |
| *gnly* | 5'- TCTCTCGTCTGAGCCC | 5'- GCAGCATTGGAAACACT |
| *id2* | 5'- CGGATATCAGCATCCTGTCC | 5'- TCATGAACACCGCTTATTCAG |
| *18s* | 5'- GGGACTTAATCAACGCAAGC | 5'- GCAATTCCCCATGAACG |
