# Supplementary figures and images for: Ionomycin Treatment Renders NK Cells Hyporesponsive
Source: PLoS One. 2016 Mar 23;11(3):e0150998. doi: 10.1371/journal.pone.0150998 (PMC4805247; doi:10.1371/journal.pone.0150998)

Supplementary Figure 1

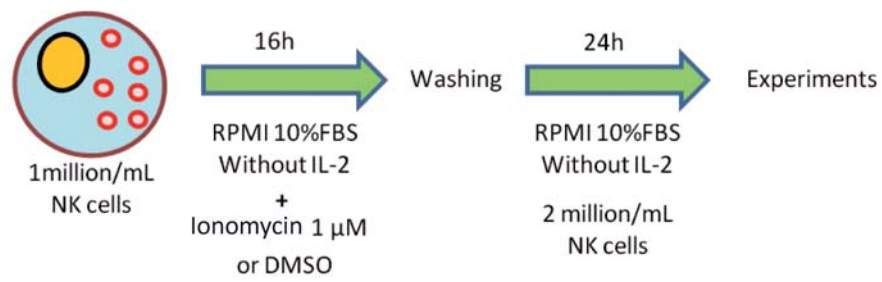

Supplement: S1 Fig — (PDF) [file pone.0150998.s002.pdf]

Supplementary FIGURE 2

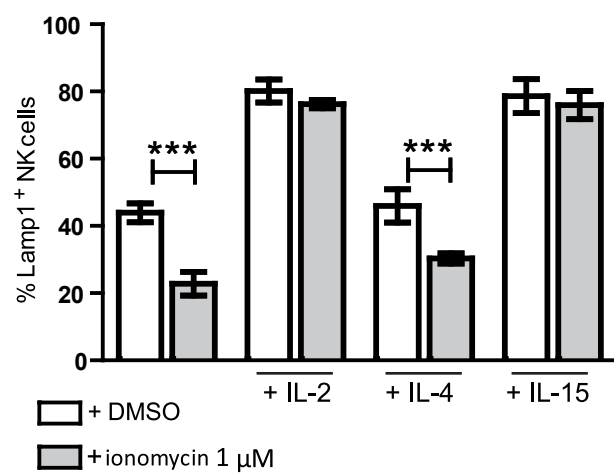

Supplement: S2 Fig — During the rest period aliquots of ionomycin treated or control cells were cultured either in medium alone, or medium supplemented with the indicated recombinant cytokines: IL-2, 50U/ml; IL-4, 1000U/ml; IL-15, 10ng/ml. After this culture the NK cells were recovered, washed counted and used in degranulation assays against K562 target cells. (n = 3). Two way paired ANOVA analysis and Bonferroni post-test. The data show mean ± SEM. ***p < 0.001. (PDF) [file pone.0150998.s003.pdf]

SUPPLEMENTARY FIGURE 3

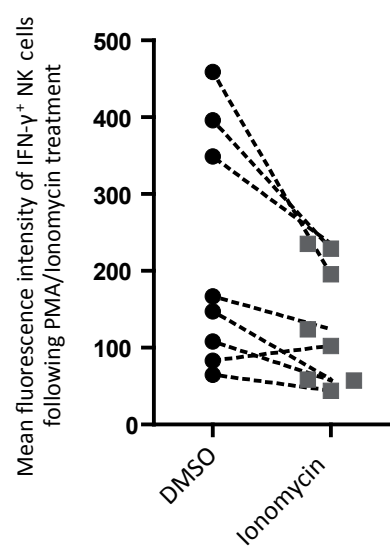

Supplement: S3 Fig — IFN-γ production by NK cells was measured by flow cytometry after a 4 hours accumulation in the presence of 2.5 μM monensin. Data shown as mean fluorescence intensity (n = 8). (PDF) [file pone.0150998.s004.pdf]

Supplementary FIGURE 4

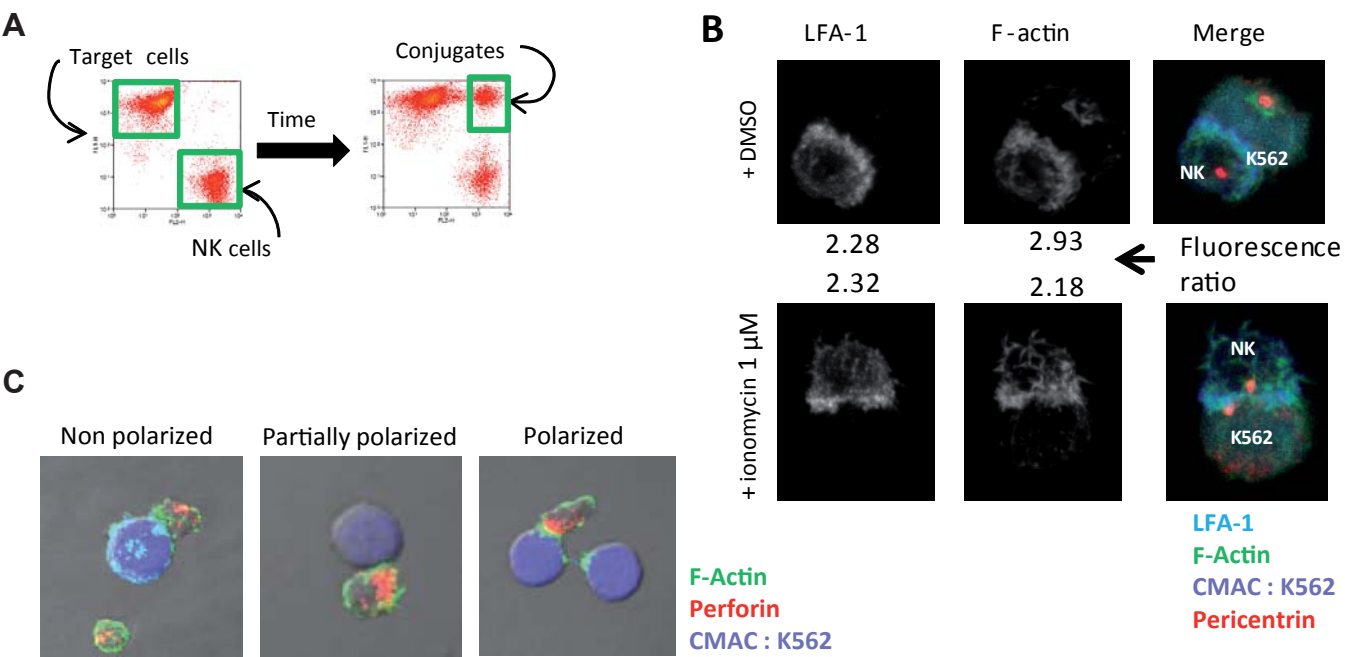

Supplement: S4 Fig — (A) Gating strategy followed to study conjugate formation between NK and K562 cells. (B) Representative examples of control (top) and ionomycin treated (lower) cells staining of single Alexa 648 (LFA-1) and Alexa 488 (F-actin) channel colors and their merge. (C) Representative example of lytic granules polarization as non-polarized, partially polarized and polarized. F-actin (Phalloidin-Alexa 488) appears in green, lytic granules in red (Perforin antibody + GAM Alexa 568I and target cells in blue (CMAC dye). (PDF) [file pone.0150998.s005.pdf]

Supplementary FIGURE 5

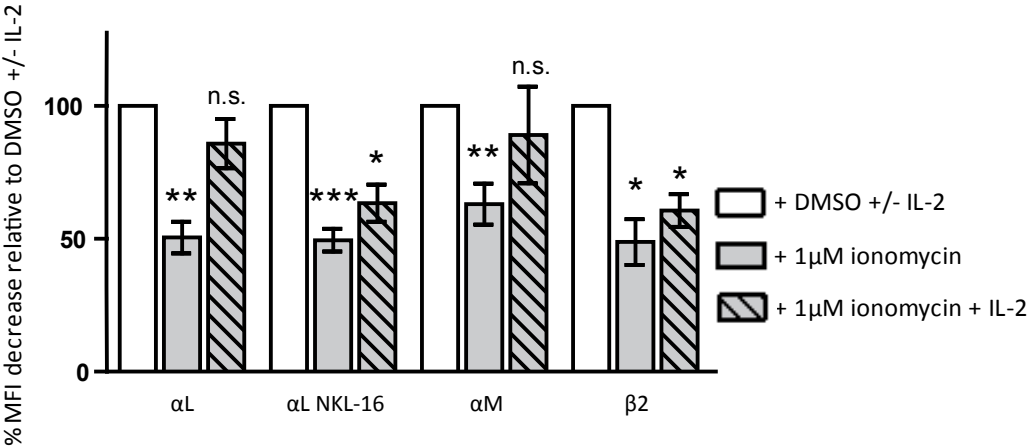

Supplement: S5 Fig — Hatched grey bars: Ionomycin treated cells stimulated with IL-2 during the rest day (n = 3–12). The data show mean fluorescence ± SEM of those experiments where integrin expression decreased. Two tailed paired Student's T test analysis of the logarithm of raw data was used. *p < 0.05, **p < 0.01, ***p < 0.001, n.s.: non-significant. (PDF) [file pone.0150998.s006.pdf]

SUPPLEMENTARY FIGURE 6

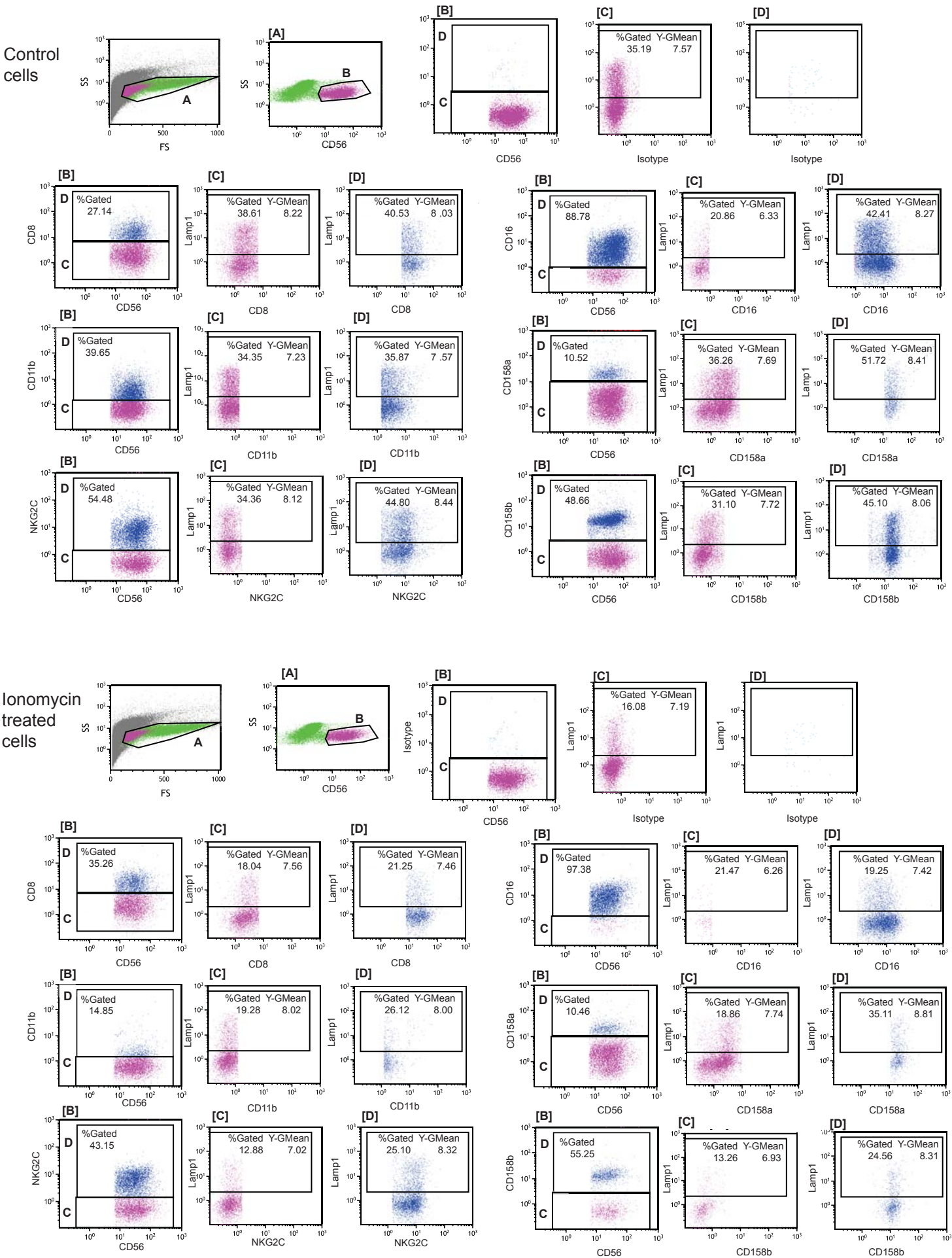

Supplement: S6 Fig — NK cells were identified as CD56 positive cells ([B] gate), and gated as marker negative ([C] gate) and positive ([D] gate. Percentage of positive cells is shown). The degranulation of cells positive and negative for each marker analysed is also shown (percentage and geometric mean of fluorescence intensity of Lamp1 positive cells). (PDF) [file pone.0150998.s007.pdf]

Supplementary Figure 8

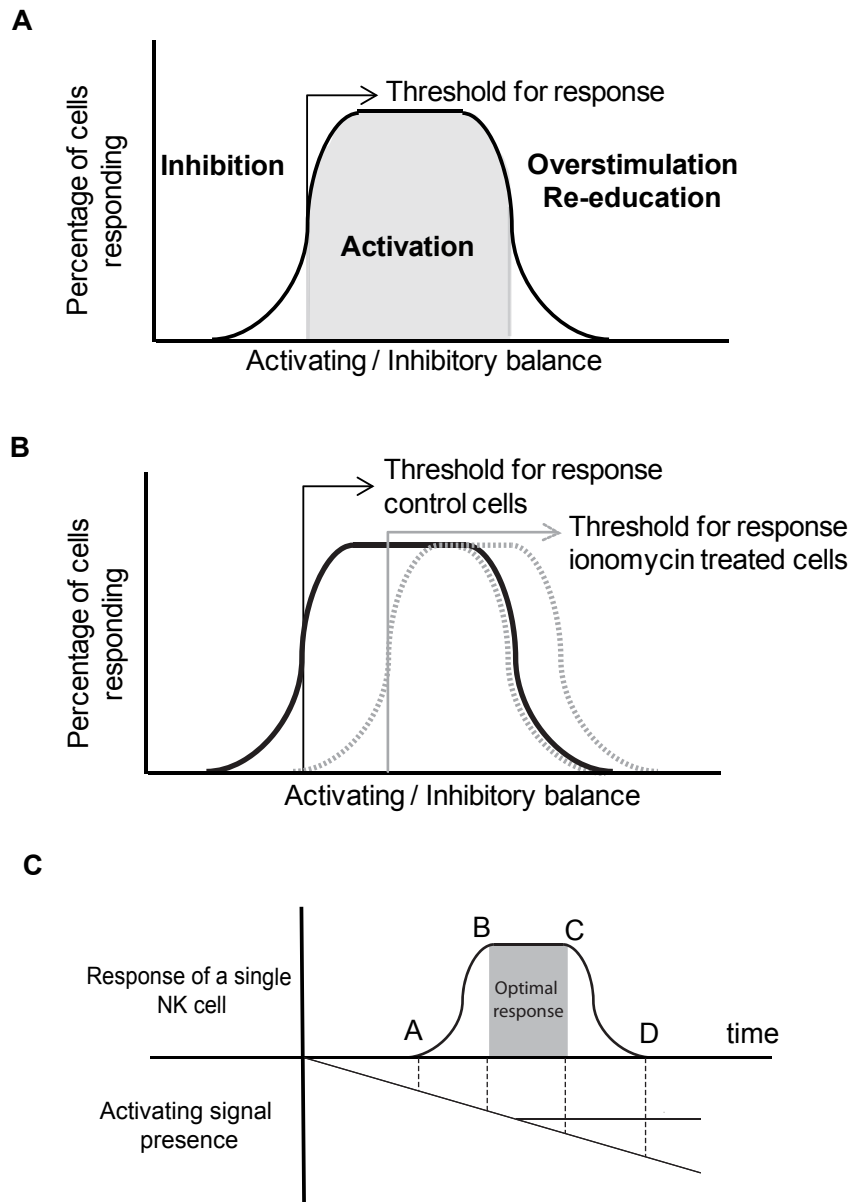

Supplement: S8 Fig — (A) The disarming model implies that during education there exists one threshold for inactivation, which if exceeded triggers NK cell hyporesponsiveness. When licensed, NK cells will have two thresholds, one for activation and another for inactivation after overstimulation. (B) Ionomycin treatment could cause a recalibration of at least the NK cell threshold for response, increasing the amount of activation signal required for an NK cell response. (PDF) [file pone.0150998.s009.pdf]
