# Supplementary material for: Ionomycin Treatment Renders NK Cells Hyporesponsive
Source: PLoS One. 2016 Mar 23;11(3):e0150998. doi: 10.1371/journal.pone.0150998 (PMC4805247; doi:10.1371/journal.pone.0150998)

Supplementary FIGURE 7

1.- Gene expression of human NK cells compared with other cell types [43]

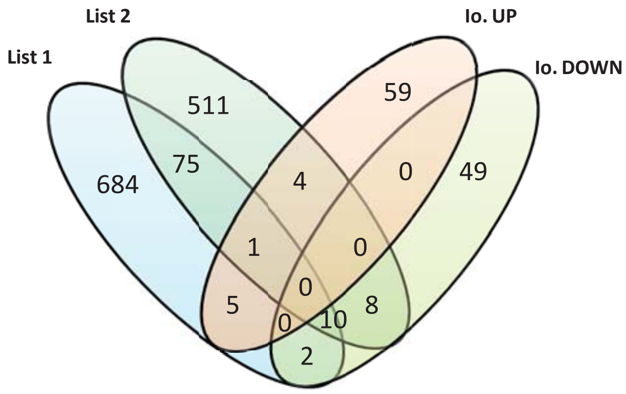

Common elements in "List 1", "List 2" and "Io. DOWN":

GZMH	ITGB2
IL18R1	CTSW
PLAC8	RGS18
GNLY	HCST
ITGAM	
CD300A	

Common elements in "List 2" and "Io. DOWN":

CCL4	NCAM1
ADRB2	PTGDR
IRF8	NCR3
EGR2	TNF

Common elements in "List 1" and "Io. DOWN":

GZMA
SLAMF8

Common elements in "List 1", "List 2" and "Io. UP":

RAMP1

Common elements in "List 2" and "Io. UP":

CD160	DUSP2
B3GNT7	DUSP4

Common elements in "List 1" and "Io. UP":

PYCR1	TMEPAI
MOXD1	NMB
GNG4	

2.- Activated CD56^{dim}CD16^{+} NK vs. CD56^{dim}CD16^{+} NK cells [44]

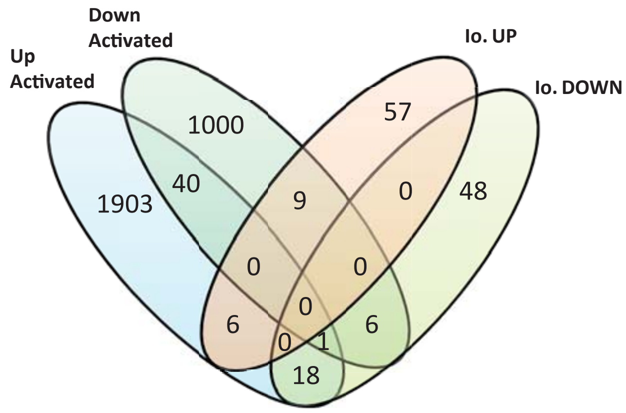

Common elements in "Up Activated" and "Io Down"

LTB	GZMH
GZMK	RNASE6
CTSW	IRF8
CD52	CCR5
MMP25	CCR1
GNLY	ITGAM
GZMA	LTC4S
XBP1	CD300A
HCST	C10orf128

Common elements in "Up Activated", "Down Activated" and "Io Down":
CCL3

Common elements in "Up Activated" and "Io. UP":

MAL	CYP1B1
FABP5	PYCR1
CD9	HSPB1

Common elements in "Down Activated" and "Io. DOWN":

NCAM1	PCSK5
GIMAP5	PLAC8
PTGDR	STOM

Common elements in "Down Activated" and "Io. UP":

HES6	RNF130
CD109	RAMP1
CD160	DUSP2
LAYN	PMEPA1
CTHRC1	

3.- Tolerant murine CD8^{+} T cells [42]

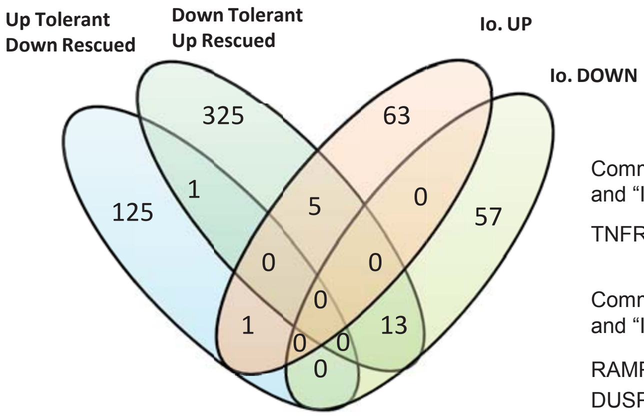

Common elements in "Up Tolerant" and "Io. UP":

TNFRSF9

Common elements in "Up Rescued" and "Io. UP".:

RAMP1
DUSP2
VCAM1
LMNA
HMOX1

Common elements in "Up Rescued and "Io. DOWN":

EGR1
EGR2
TNF
GZMK
RNASE6
CCR5
IL18R1
CTSW
NT5E
PLAC8
CXCR6
HCST
CCL4

4.- Murine HIV exhausted CD8$^+$ T cells

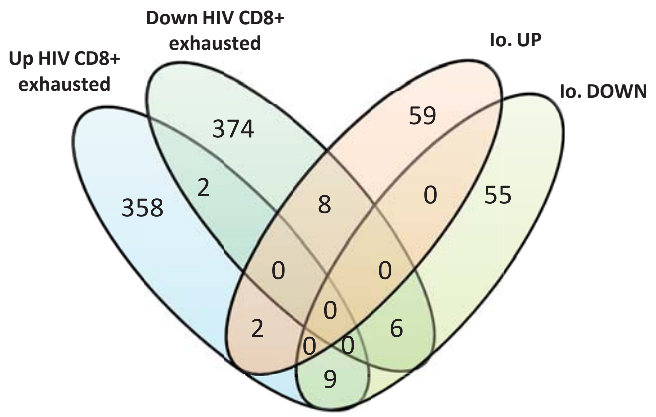

Common elements in "Up Exhausted" and "Io. UP":

TNFRSF9
IGJ

Common elements in "Up Exhausted" and "Io. DOWN":

IFITM1
STOM
CCL3
CCL3L3
GZMA
EGR1
CCR5
GZMH
CCR1

Common elements in "Down Exhausted" and "Io. DOWN":

TIMP1
IL18R1
ITGAM
TNF
SLAMF8
PCSK5

Common elements in "Down Exhausted" and "Io. UP":

CYP1B1
SLC7A5
MAL
LEF1
LMNA
CD9
NBL1
DUSP4

5.- Exhausted CD8$^+$ T cells from mice chronically infected with LCMVclone13 *vs*. effector CD8$^+$ T cells

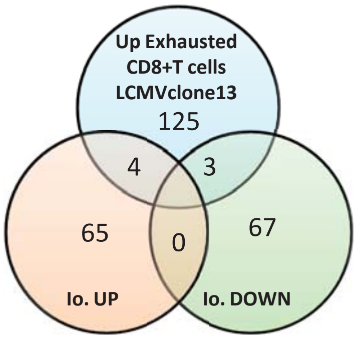

Common elements in "Up Exhausted CD8+" and "Io. UP":

CD160
TNFRSF9
VCAM1
CD9

Common elements in "Up Exhausted CD8+" and "Io. DOWN

ITGB2
CCR2
EGR2

6.- Ionomycin induced anergic CD4$^+$ T cells

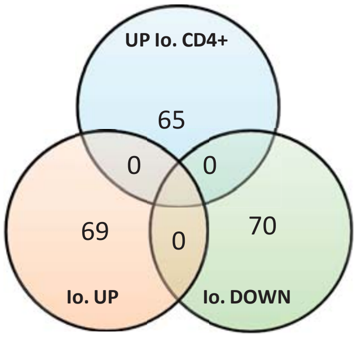

Supplement: S7 Fig — (PDF) [file pone.0150998.s008.pdf]
