# Supplementary material for: Ionomycin Treatment Renders NK Cells Hyporesponsive
Source: PLoS One. 2016 Mar 23;11(3):e0150998. doi: 10.1371/journal.pone.0150998 (PMC4805247; doi:10.1371/journal.pone.0150998)
Supplement: S1 Table — (PDF) [file pone.0150998.s010.pdf]

SUPPLEMENTARY TABLE 1

Fold Change	Gene Name
12,44	**CTHRC1**
11,08	**PLS3**
9,1	**KIAA1324**
7,24	**CD27**
5,61	**PON3**
5,32	**CD9**
5,02	**EPAS1**
4,66	**CAV1**
4,45	**C7orf57**
4,27	**TNFRSF9**
4,21	**GNG4**
4,2	**NUAK1**
4,15	**NMB**
4,01	**IGJ**
3,97	**CYP1B1**
3,94	**HMOX1**
3,69	**REEP2**
3,53	**P2RX5**
3,5	**ITGB5**
3,5	**P2RX5**
3,47	**HPSE**
3,45	**CD160**
3,41	**HES6**
3,39	**CREB3L3**

Fold Change	Gene Name
3,34	**VCAM1**
3,28	**FABP5**
3,26	**LMNA**
3,24	**FLVCR2**
3,23	**B3GNT7**
3,22	**PLAU**
3,19	**ZBED2**
3,11	**RNF130**
3,05	**CBFA2T3**
2,94	**HSPB1**
2,92	**MAL**
2,9	**MOXD1**
2,89	**RDH10**
2,87	**LAYN**
2,75	**TOP1MT**
2,72	**DUSP2**
2,71	**UCHL1**
2,64	**PYCR1**
2,62	**IGFBP2**
2,62	**PMCH**
2,59	**TMEM117**
2,59	**CD109**
2,58	**DMRT1**
2,57	**COL6A1**

Fold Change	Gene Name
2,56	**MAGEF1**
2,53	**BMF**
2,52	**HOXB6**
2,49	**SLC7A5**
2,49	**PPAP2A**
2,48	**CERCAM**
2,44	**PTPRF**
2,41	**DUSP4**
2,41	**NBL1**
2,4	**LEF1**
2,39	**RAMP1**
2,34	**PRKCDBP**
2,27	**TUBB2A**
2,25	**PRKCE**
2,25	**LRRC28**
5,17	**A_23_P317056**
3,57	**LRRC16B**
2,8	**A_23_P431853**
2,69	**PMEPA1**
2,6	**A_24_P272515**
2,6	**CR591103**
2,56	**A_33_P3253832**
2,3	**LOC100008589**
1,56	**A_24_P307384**

Fold Change	Gene Name
-4,81	**CD300C**
-4,69	**ZNF683**
-4,64	**EGR1**
-4,6	**CCL3L3**
-4,46	**CCR2**
-4,29	**IRF8**
-4,24	**CD300A**
-4,03	**SELL**
-3,95	**HS3ST3B1**
-3,82	**MATK**
-3,62	**CCL3**
-3,49	**CCL4**
-3,46	**CCL4L1**
-3,4	**XBP1**
-3,36	**PTGDR**
-3,3	**STYK1**
-3,25	**GZMK**
-3,1	**TNF**
-3,07	**ITGB2**
-3	**CCDC65**
-2,98	**CTSW**
-2,98	**GZMA**
-2,97	**GNLY**
-2,94	**EGR2**
-2,81	**CD52**

Fold Change	Gene Name
-2,81	**RNASE6**
-2,8	**NCF2**
-2,77	**RGS18**
-2,76	**ITGAM**
-2,74	**LTC4S**
-2,74	**MYOM2**
-2,74	**HCST**
-2,71	**PACSIN1**
-2,68	**CCR1**
-2,67	**GZMH**
-2,67	**NCAM1**
-2,67	**CXCR6**
-2,65	**CCR5**
-2,63	**LTB**
-2,63	**SPRY2**
-2,57	**SNCA**
-2,53	**ADRB2**
-2,53	**IL3RA**
-2,47	**CCR3**
-2,47	**C20orf197**
-2,46	**CMKLR1**
-2,46	**TSPAN32**
-2,4	**CXCR7**
-2,38	**C10orf128**
-2,35	**GPD1L**

Fold Change	Gene Name
-2,35	**PLAC8**
-2,34	**TIMP1**
-2,3	**NT5E**
-2,27	**ITGA6**
-2,27	**APOBEC3H**
-2,27	**NCR3**
-2,24	**SLAMF8**
-2,23	**STOM**
-2,23	**IL18R1**
-2,21	**PCSK5**
-2,2	**CPNE7**
-2,2	**MMP25**
-2,17	**GIMAP5**
-3,71	**A_33_P3397473**
-4,49	**ENST00000390595**
-4,25	**ENST00000390605**
-4,45	**ENST00000390622**
-2,72	**ENST00000400702**
-4,61	**ENST00000443661**
-2,22	**IFITM1**
-4,48	**LOC100133862**
-3,18	**LOC100290415**
-2,97	**LOC100292999**
-2,65	**RGL4**
-2,44	**UNQ6228**
