# Supplementary material for: Ionomycin Treatment Renders NK Cells Hyporesponsive
Source: PLoS One. 2016 Mar 23;11(3):e0150998. doi: 10.1371/journal.pone.0150998 (PMC4805247; doi:10.1371/journal.pone.0150998)
Supplement: S2 Table — (PDF) [file pone.0150998.s011.pdf]

SUPPLEMENTARY TABLE 2

**Ionomycin downregulated genes**	**ENRICHED PATHWAYS**	
	**defense responses** (GO:0006952)	*gimap5, mmp25, il18r1, ncr3, nt5e, ccr3, il3ra, adrb2, snca, ccr5, ccr1, ncf2, rnase6, gnly, itgb2, tnf, ccl4l1, ccl4, ccl3, ccr2*
	**inflammatory response** (GO:0006954)	*mmp25, ncr3, nt5e, ccr3, il3ra, adrb2, ccr5, ccr1, itgb2, tnf ,ccl4l1, ccl4, ccl3, ccr2*
	**regulation of immune system process** (GO:0002682)	*egr1, gimap5, ncr3, snca, tnf*
	**innate immune response** (GO:0045087)	*ncam1, ltb, adrb2, tnf*
	**chemotaxis** (GO:0006935)	*ccr2, ccl3, ccl4, ccl4l1, itgb2, itgam, ccr1, ccr5, ccr3, cmklr1*
	**locomotory behavior** (GO:0007626)	*cmklr1, itgam, ccr3, snca, ccr5, ccr1, itgb2, ccl4l1, ccl4, ccl3, ccr2*
	**cellular calcium ion homeostasis** (GO:0006874)	*ccr2, ccl3, cd52, ccr1, ccr5, ccr3*
	**cell-cell signaling** (GO:0007267)	*pcsk5, tspan32, spry2, egr2, ltb, egr1, snca, ccr5, ccr1, itgb2, tnf, ccl4, ccl3*
	**cell adhesion** (GO:0007155)	*itga6, cd300a, sell, ncam1, itgam, ccr3, ccr1, itgb2, tnf, ccl4*
	**integrin-mediated signaling pathway** (GO:0007229)	*ncam1, ltb, adrb2, tnf*
	**cell activation** (GO:0001775)	*ltb, itgam, egr1, gimap5, snca, tnf*
	**leukocyte activation** (GO:0045321)	*itgam, egr1, gimap5, snca*
	**immunesystem development** (GO:0002520)	*timp1, irf8, ltb, egr1, gimap5, tnf*
	**regulation of cell proliferation** (GO:0042127)	*ifitm1, timp1, ltb, adrb2, tnf*
	**cytokine production** (GO:0001816)	*pcsk5, ltb, gimap5, tnf*
	**positive regulation of signal transduction** (GO:0009967)	*ncam1, ltb, adrb2, tnf*
	**positive regulation of programmed cell death** (GO:0043067)	*ncam1, ltb, adrb2, tnf*
	**ENRICHED CELLULAR LOCALIZATIONS**	
	**cell surface** (GO:0009986)	*ITGA6, ccr5, ncam1, itgam, sell, tnf, mmp25*
	**membrane rafts** (GO:0045121)	*stom, adrb2, tnf*
	**extracellular space** (GO:0005615)	*ltb, gnly, tnf, ccl4l1, ccl4, ccl3, ccl3l3, pcsk5*
	**ENRICHED FUNCTIONS**	
	**cytokine activity** (GO:0005125)	*ltb, tnf,* ENST00000400702, *ccl4l1, ccl4, ccl3, ccl3l3*
	**cytokine binding** (GO:0019955)	*il18r1, il3ra, ncam1, cxcr7, cmklr1, ccr3, ccr5, cxcr6, ccr1, ccr2*
	**peptidase activity** (GO:0008233)	*mmp25, gzmh, ctsw, gzmk, gzma, pcsk5*
	**cell death** (GO:0008219)	*ltb, gzmh, gzma, itgb2, tnf*
	**natural killer cell mediated cytotoxicity** (hsa04650)	*ncr3, hcst, itgb2, tnf*
	**cytokine-cytokine receptor interaction** (hsa04060)	*il18r1, il3ra, ltb, ccr5, cxcr6, ccr1, tnf, ccl4l1, ccl4, ccl3, ccr2, ccl3l3*
	**leukocyte transendothelial migration** (hsa04670)	*nf2, itgam, itgb2*
**Ionomycin upregulated genes**	**ENRICHED PATHWAYS**	
	**response to organic substance** (GO:0010033*)*	*creb3l3, cyp1b1, lef1, ptprf, igfbp2, gng4, dusp4, cd27, cav1, hspb1, hmox1*
	**regulation of signal transduction** (GO:0009966)	*lef1, ptprf, igfbp2, p2rx5, gng4, ramp1, dusp2, cd27, cav1, hmox1*
	**positiveregulation of signal transduction** (GO:0009967)	*p2rx5, cd27, cav1, hmox1*
	**positive regulation of programmed cell death** (GO_0043068)	*ptprf, prkce, mal, tnfrsf9, cd27, hmox1*
	**dephosphorylation** (GO:0016311)	*ptprf, prkce, mal, tnfrsf9, cd27, hmox1*
	**cellular ion homeostasis** (GO:0006873*)*	*pmch, p2rx5, mal, cav1*
	**ENRICHED CELLULAR LOCALIZATIONS**	
	**insoluble fraction** (GO:0005626)	*hspb1, lmna, prkce, ptprf, ppap2a, rdh10, mal, hmox1, cyp1b1, cav1, pon3, cd27*
	**extracellular space** (GO:0005615)	*ramp1, cd109, pmch, igfbp2, vcam1, hmox1, pon3*
	**vesicular fraction** (GO:0042598)	*ptprf, rdh10, hmox1, cyp1b1, pon3*
	**ENRICHED FUNCTIONS**	
	**phosphatases** (GO:0016791)	*dusp4, ptprf, ppap2a, dusp2, pon3*
	**oxidoreductases** (GO:0016491)	a_23_p431853, a_23_p317056, *igfbp2, pycr1, rdh10, moxd1, cyp1b1, hmox1*
